# Ultra-long-term subcutaneous EEG recordings in ten epilepsy patients: Experiences and circadian rhythms in epileptiform discharges

**DOI:** 10.1016/j.ebr.2025.100781

**Published:** 2025-05-20

**Authors:** S.J. van Norden, K.H. Kho, A.M. Meppelink, J.J. Ardesch, M.C. Tjepkema-Cloostermans, M.J.A.M. van Putten

**Affiliations:** a Departments of Clinical Neurophysiology and Neurology, Medisch Spectrum Twente (MST), Koningstraat 1, 7512KZ Enschede, the Netherlands; b Department of Clinical Neurophysiology, University of Twente (UT), Drienerlolaan 5, 7522NB Enschede, the Netherlands; c Department of Neurosurgery, Medisch Spectrum Twente (MST), Koningstraat 1, 7512KZ Enschede, the Netherlands; d Stichting Epilepsie Instellingen Nederland (SEIN), Dokter Denekampweg 20, 8025BV Zwolle, the Netherlands

**Keywords:** Pharmcoresistant epilepsy, Ultra-long-term EEG, Subcutaneous EEG, Recording compliance, Epileptiform discharges

## Abstract

Recently, a subcutaneous electroencephalography (sqEEG) electrode became available, enabling ultra-long-term EEG recordings. In our PREDYct study, we aim to predict the efficacy of vagus nerve stimulation (VNS) in epilepsy patients. The study records sqEEG for up to fifteen months, including a 2–3 month period before VNS implantation. Here we report on initial patient experiences, compliance and recordings of epileptiform discharges (EDs). We included the first 10 participants from our ongoing PREDYct study. We calculated recording compliance for each patient. Patients rated their pain during electrode implantation, wearing and explantation using the visual analogue scale (VAS). Approximately two weeks of data from each patient were visually assessed and all EDs were annotated. We used these annotations to evaluate circadian rhythms in ED rate and duration and to compare seizure diaries with annotated ictal patterns. Electrode implantation and explantation were well-tolerated with a median VAS of 4 and 2, respectively. Wearing the electrode was also well-tolerated with median VAS scores of 0 and 0.5 at two and fifteen months, respectively. On average, patients wore the electrode for 441 days with an overall compliance of 27 %, resulting in 28,331 h of EEG. A circadian rhythm in ED rate was observed in three patients, while one patient exhibited a circadian rhythm in ED duration. A comparison of reported seizures and annotated ictal patterns revealed discrepancies. Ultra-long-term sqEEG is well-tolerated and has the potential to be a valuable tool for monitoring treatment. Nevertheless, it remains a challenge but crucial to attain high recording compliance.

## Highlights

• EEG electrode implantation, explantation, and continuous wear were well tolerated.
• On average, the electrode was worn for 441 days, with an overall compliance of 27 %.
• A circadian rhythm in epileptiform discharge rate was observed in three patients.
• Reported seizures and annotated ictal patterns revealed discrepancies.

## 1 Introduction

Epilepsy is a neurological condition in which the brain is prone to generate recurring epileptic seizures [1]. Anti-seizure medication (ASM) is the initial treatment for epilepsy, primarily aimed at reducing the patient’s seizure frequency. In cases where ASM is not sufficient, surgical removal of the epileptic zone can be considered for those with focal epilepsy [2,3]. Patients with pharmacoresistant epilepsy who are not eligible for surgical treatment may benefit from therapy with deep brain stimulation (DBS) or vagus nerve stimulation (VNS). Reported neurostimulation responder rates, defined as a reduction in seizure frequency of at least 50 %, range from 56 to 74 % [4]. An insufficient effect by neurostimulation is not only disappointing for the patient and involved clinicians, but there are also significant healthcare costs involved.

In general, assessing the efficacy of treatment in epilepsy is constrained by the quality of our readouts as we essentially depend on patient-reported seizure diaries, despite their limited reliability. [5,6]. Both under- and overreporting of seizures occur regularly using seizure diaries [5,7,8]. Nocturnal seizures and difficult-to-recognize seizures are most likely to be underreported, with 86 % and 20–73 % of seizures unreported, respectively [7]. These results were confirmed by a study involving 3407 patients, which also revealed that 58 % of documented seizures were actually non-correlated events, showing no clinical change or alterations in EEG [9]. The reliance on seizure diaries may result in under- or overtreatment of patients, and may also affect reported outcomes in epilepsy research [10,11].

Lately, a new generation of EEG devices has emerged, enabling the recording of EEG during everyday life [[12], [13], [14], [15]]. A notable example is minimally invasive subcutaneous EEG (sqEEG) as recorded with the 24/7 EEG*^TM^* SubQ (UNEEG medical A/S, Lynge, Denmark) [5,14]. This system allows for unilateral two-channel EEG recordings for up to fifteen months [14], providing a reliable readout for seizures, that may eventually make seizure diaries less crucial [5,6].

In 2020, we initiated a prospective observational cohort study (’Predicting efficacy of neuromodulation in epilepsy’, PREDYct) in epilepsy patients who were candidates for treatment with VNS. In the PREDYct study, patients are implanted with the sqEEG electrode two months prior to VNS implantation, aiming to identify biomarkers that predict VNS efficacy. After VNS implantation, sqEEG recordings continue for up to fifteen months, allowing us to assess the effects of VNS on seizures. We aim to include 40 patients in the PREDYct study.

In this preliminary analysis, our aim is to evaluate patients’ experiences with long-term sqEEG recordings and to explore the occurrence and temporal distribution of epileptiform discharges (EDs) in this unique observational cohort. Notably, patients in this study do not directly benefit from the sqEEG recordings, offering a valuable opportunity to study adherence and EEG patterns outside of a clinical monitoring context. We were particularly interested in identifying potential circadian patterns in EDs, an area that has received relatively little attention compared to seizure-focused ultra-long-term monitoring studies [5,8].

## 2 Methods

### 2.1 PREDYct study

We used data from patients with frontotemporal or generalized epilepsy who were candidates for VNS implantation and enrolled in our ongoing ’Predicting efficacy of neuromodulation in epilepsy’ (PREDYct) study. The study is performed at Medisch Spectrum Twente (MST, Enschede, the Netherlands), in collaboration with the University of Twente (UT, Enschede, the Netherlands) and Stichting Epilepsie Instellingen Nederland (SEIN, Zwolle, the Netherlands). The study was approved by the METC (MEC-U; reference number NL73089.100.20) and the local institutional review board (study number H20-06). All patients gave written informed consent.

**Fig. 1:**
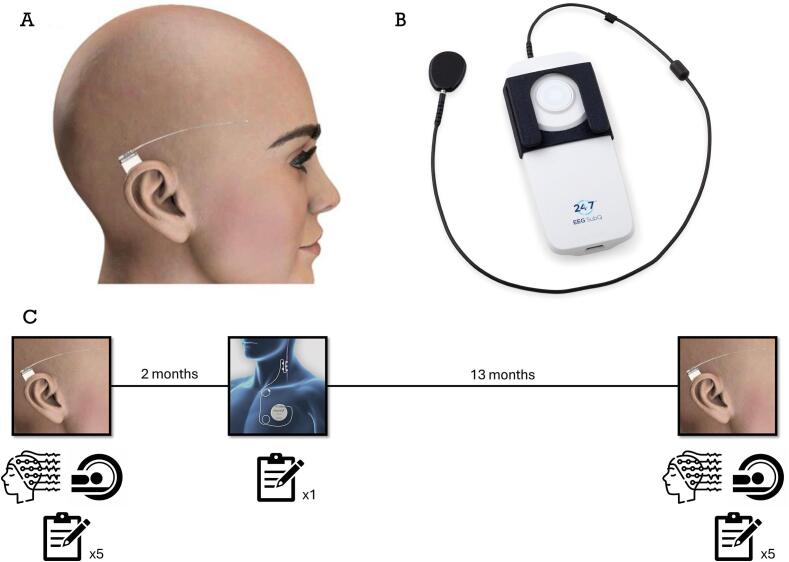
**The 24/7 EEG*^TM^* SubQ and its use in the PREDYct study.** A) The subcutaneous EEG electrode, consisting of three leads, positioned over the frontotemporal area. B) The external logging device. C) Timeline of the PREDYct study highlighting the three key time points: electrode implantation, VNS implantation and electrode explantation. At electrode implantation and explantation, a scalp EEG and an MRI scan were conducted and five questionnaires were completed. At VNS implantation, one questionnaire was completed. Patients were encouraged to wear the logging device from one to two weeks after electrode implantation until electrode explantation.

Included patients were implanted with a subcutaneous EEG electrode (part of the EEG*^TM^* SubQ, UNEEG medical A/S, Lynge, Denmark) two months prior to VNS surgery. The electrode was positioned horizontally over the frontotemporal area above the ear, as illustrated in Fig. 1A. For patients with focal seizures, the side of implantation was based on the origin of the interictal discharges assessed with formerly recorded routine scalp EEG and clinical evidence indicating the hemisphere from which the seizures originate [14,16]. In patients with generalized seizures, the side of implantation was based on their preference.

The sqEEG electrode (Fig. 1A) consists of three leads (a distal (D*_SQ_*), proximal (P*_SQ_*) and center (C*_SQ_*) lead), allowing two bipolar recordings from channels D*_SQ_*-C*_SQ_* and C*_SQ_*-P*_SQ_*. Besides the electrode, the system consists of a transceiver which is connected to a logging device (Fig. 1B) that stores data sampled at approximately 207 Hz. One to two weeks after electrode implantation, the transceiver was attached to the skin with double-sided adhesive pads to start EEG recordings. Patients were instructed to change the logging device daily to recharge it and to transfer the data to a USB stick using a laptop with specialized software (UNEEG Home Data Manager System). This USB stick was transferred to the MST via regular post every two months. Patients were provided with feedback on their compliance each time we received a USB stick. In case of low or declining compliance, we aimed to improve patient adherence by identifying potential barriers and offering individualized support. With patient consent, home visits were conducted as an additional strategy to improve compliance. In case we suspected a drop in compliance in between these two months, the patient was contacted with greater frequency. Ultra-long-term EEG recordings continued for up to fifteen months.

On the day of electrode implantation and explantation, we also recorded a 30-minute 64-channel EEG, performed an MRI scan (anatomical, diffusion weighted imaging (DWI) and functional MRI (fMRI)) and took four well-being questionnaires. The 64-channel EEGs and MRIs have not been analyzed in this paper. The well-being questionnaires include Dutch translations of the Beck Depression Inventory-II (BDI-II), the General Anxiety Disorder-7 (GAD-7), the Patient Health Questionnaire-9 (PHQ-9) and the Patient Weighted Quality of Life in Epilepsy (QOLIE-31-P) [[17], [18], [19], [20]]. Patients were asked to rate their pain during the electrode implantation and explantation procedure on a 0–10 visual analogue scale (VAS) and to comment on their experience during the procedures. Approximately two (VNS implantation) and fifteen (electrode explantation) months following electrode implantation, patients completed a questionnaire about their experience wearing the 24/7 EEG*^TM^* SubQ, including a 0–10 VAS for pain and comments regarding their overall experience. Patients were asked to maintain a daily seizure diary throughout their participation in the PREDYct study. Patients who were already maintaining a seizure diary, either online or on paper, were instructed to continue doing so. For those who did not, a paper diary was provided.

The VNS was activated at SEIN approximately one month after VNS implantation. In the months following, VNS parameter settings were adjusted according to the patients seizure diary and experienced side effects. During these visits, efforts were made to encourage patients to wear the logging device as frequently as possible and to maintain their seizure diary. An overview of the timeline of the PREDYct study is shown in Fig. 1C.

Here, we focus on patients’ experiences with the 24/7 EEG*^TM^* SubQ, including VAS scores and sqEEG recording compliance, and on our findings in the first two weeks of sqEEG recordings.

### 2.2 Data analysis

#### 2.2.1 Patient compliance

We calculated recording compliance for each patient based on the amount of data that was recorded between the start of the EEG recordings (one to two weeks after electrode implantation) and electrode explantation. The correlation between recording compliance and VAS scores after wearing the device for two and fifteen months, as well as the correlation between recording compliance and the scores from the four well-being questionnaires (both pre and post VNS), were calculated using a linear mixed model.

#### 2.2.2 Epileptiform discharges

The first two weeks of data from each patient were visually assessed and IEDs and ictal patterns were annotated by an experienced neurologist/clinical neurophysiologist (MvP). If recording compliance was low during the initial two weeks, additional recordings were annotated to approximate two full weeks of data. The raw EEG data were evaluated in 10-second epochs for annotation. We calculated the hourly ED rate and mean hourly ED duration from the annotated data using MATLAB R2024A (The MathWorks, Inc., Natick, MA). The ED rate was adjusted for missing data based on hourly recording compliance. For example, if the compliance in a specific hour was 90 %, the ED rate was multiplied by 100/90.

We used Lomb-Scargle power spectral densities (PSDs) to assess circadian rhythms in ED rate and mean ED duration [21,22]. The ED rate and ED duration were therefore normalized by centering the mean to 0 and scaling by the standard deviation. We calculated the 0.1 % false-alarm probability to ascertain the significance of peaks in the PSD. The 0.1 % false alarm probability threshold was calculated analytically under the null hypothesis that the data consists of pure white noise. In this case, the test statistic—normalized spectral power at each frequency—follows an exponential distribution, allowing the threshold to be set based on the probability of observing a peak by chance [23,24]. In case a significant circadian rhythm was found, we computed the phase preference with its phase locking value (PLV) after bandpass filtering (0.8–1.2 days). The PLV is defined as

(1) $$\mathrm{PLV}=\frac{1}{N}\left|\sum e^{\phi -\phi ref}\right|,$$

where *N* represents the number of hours in the analyzed data segment, *ϕ* represents the instantaneous phase of the smoothed ED rate or ED duration and *ϕ_ref_* represents the instantaneous phase of a sine wave with a period of 24 h, serving as a reference for the circadian cycle. We included only data segments of at least 36 consecutive hours for PLV calculation. When multiple segments were available for a patient, we computed the PLV for each segment and then averaged them, weighting each PLV by the duration of its respective segment. For instance, if a patient had one segment of 40 h and another of 80 h, the PLV from the 80-hour segment contributed twice as much to the final average as the 40-hour segment.

The ED rate and average ED duration were visualized after applying a 3-hour moving average. We visualized the circadian distribution, including standard error of the mean (SEM), of the ED rate and average ED duration on a 24-hour time axis for each patient.

Finally, we compared the annotated data with patients’ seizure diaries. Here, we identified a cluster of ictal patterns as ictal patterns that occurred within 10 min of each other. We also included the recording compliance during the annotated period. This compliance was calculated in two ways: first, over the entire annotated period, and second, only for days with recorded data.

## 3 Results

At the time of this analysis, 17 patients have been enrolled in the PREDYct study. This preliminary report includes data from the first ten patients. Patient demographics of these ten patients are shown in Table 1.

**Table 1:** **Patient demographics.** Age in 5-year range. Etiology classification as defined by [25]. The duration indicates the number of years since the onset of epilepsy at the time of electrode implantation.

No.	**Sex**	**Age** **(y)**	**Type of seizures**	**Etiology**	**Duration** **(y)**	**Implantation side**
1	F	20–24	Generalized	Genetic	4	Right
2	F	20–24	Generalized	Genetic	4	Left
3	M	40–44	Generalized and focal	Unknown	17	Left
4	M	50–54	Generalized	Genetic	38	Left
5	F	25–29	Focal	Infectious	3	Right
6	M	50–54	Focal	Structural	3	Left
7	M	35–39	Generalized	Genetic	19	Left
8	F	30–34	Generalized	Genetic	23	Right
9	F	35–39	Focal	Genetic	5	Right
10	M	20–24	Generalized	Genetic	22	Right

### 3.1 Patient experience

**Fig. 2:**
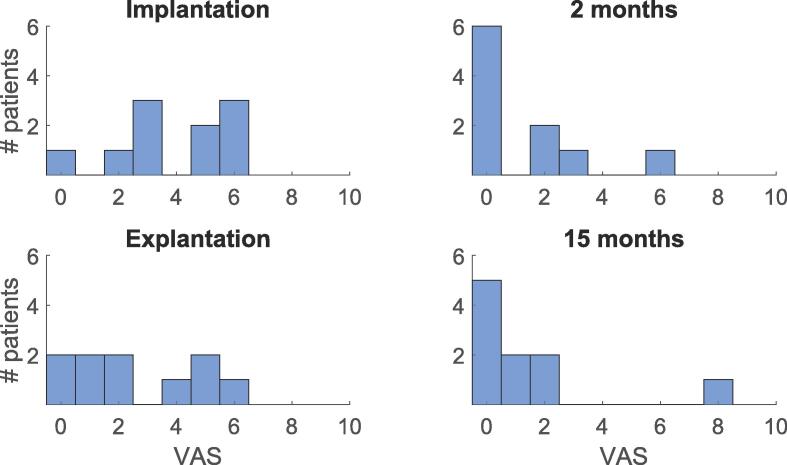
**VAS scores.** An overview of the VAS scores for electrode implantation and explantation and for wearing the electrode for two and fifteen months.

The median VAS during electrode implantation was 4, ranging from 0 to 6, and the median VAS during explantation was 2, ranging from 0 to 6 (Fig. 2). The majority of patients perceived the administration of the anaesthetic as the most painful aspect of the procedures. Following implantation, a significant proportion of patients reported discomfort due to the sound generated while inserting the electrode.

Wearing the electrode was well tolerated by nine out of ten patients with a median VAS of 0, ranging from 0 to 6, after two months and a median VAS of 0.5, ranging from 0 to 8 after fifteen months (Fig. 2). The VAS score of 8 after wearing the electrode for fifteen months indicates that one patient did not tolerate the electrode well. However, there was a discrepancy in the reported scores for this patient. During the explantation the patient reported moderate pain, yet the VAS score was 0. Conversely, when wearing the electrode, the patient reported minimal pain, yet the VAS score was 8. Several patients reported discomfort with the wire that is connecting the logging device to the transceiver. Some patients suggested the wire should be shorter and others suggested the wire should be longer, but they all agreed that a wireless solution would be optimal. Additionally, patients expressed frustration with the sound and vibration generated by the logging device when the connection was lost, particularly at night.

### 3.2 Patient compliance

**Fig. 3:**
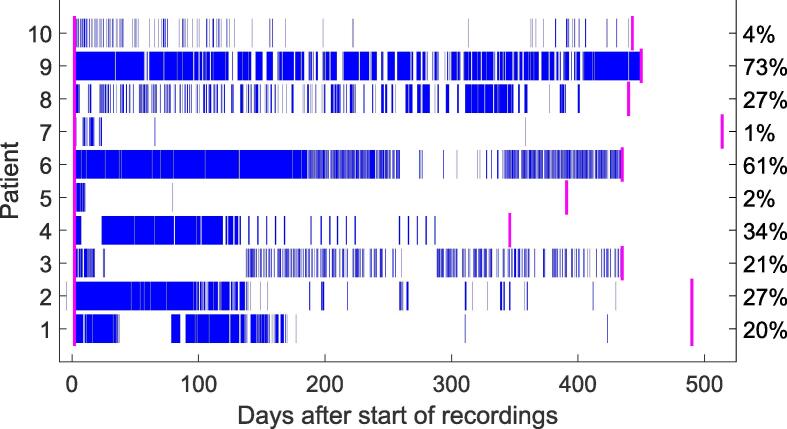
**Patient compliance.** Recorded sqEEG is shown in blue; the start of the recordings and electrode explantation are indicated in pink. We calculated the compliance based on the amount of recorded data between the two pink marks. Patients 1, 3 and 4 paused their recordings at the start of the study due to skin irritation. Several patients experienced a decline in motivation as the study progressed.

On average, patients wore the electrode for 441 days, ranging from 344 to 512 days. The median recording compliance was 24 % with a range of 1 to 73 % per patient (overall compliance of 27 %), resulting in a total of 28,331 h of data. For three patients, compliance was low in the initial weeks after electrode implantation due to skin irritation at the implantation site. Another significant factor contributing to low compliance was patient motivation. Fig. 3 illustrates patient compliance over the course of the study, showing that more recordings were made at the beginning. Patient 3 recorded EEG for a few nights early in the study after which he only recorded during the day. He noted that the sound and vibration of the logging device woke him up every night, which was a source of annoyance. At the final stage of the study, patient 4 recorded EEG for 24 h per week, primarily due to motivational issues. We did not find any significant correlations between the compliance and VAS scores or well-being questionnaires.

### 3.3 Epileptiform discharges

**Fig. 4:**
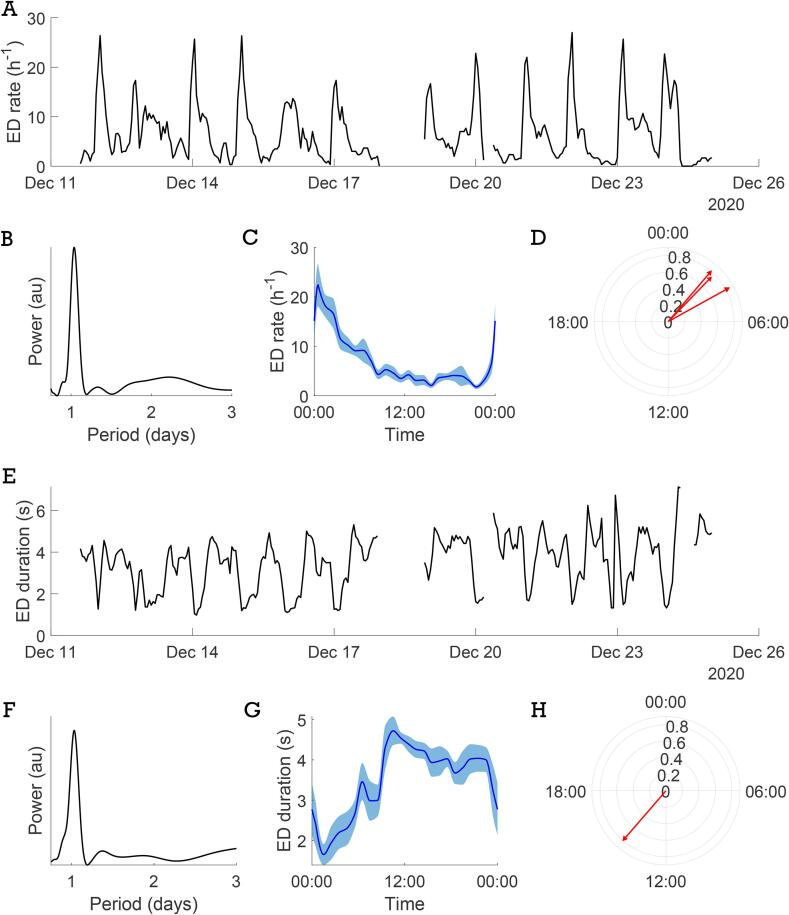
**Circadian rhythms.** Visualization of circadian rhythms in ED rate (A-D) and mean ED duration (E-H). Examples of A) the ED rate over time in patient 1, B) the Lomb-Scargle PSD of the ED rate in patient 1 and C) the circadian distribution for patient 1, together with D) an overview of phase preference and PLV for all three patients with a significant circadian rhythm in ED rate (patients 1, 4 and 7). Examples of E) the ED duration over time in patient 1, F) the Lomb-Scargle PSD of the ED duration in patient 1 and G) the circadian distribution for patient 1, together with H) an overview of phase preference and PLV for the one patient with a significant circadian rhythm in ED duration (patient 1).

On average, we annotated 253 h of data per patient, ranging from 150 to 374 h. The number of annotated EDs ranged from 5 to 3144 per patient. A circadian rhythm in ED rate was observed in three out of ten patients (Fig. 4A-D); one out of ten patients exhibited a circadian rhythm in mean ED duration (Fig. 4E-H). For patient 1, the patient in whom we identified a circadian rhythm in both ED rate and ED duration, we observed an anticorrelation between the ED rate and duration, with more frequent but shorter EDs occurring at night. For patient 10, we did not observe a circadian rhythm in ED rate but a rhythm with a period of approximately 1.5 days was noted.

We found discrepancies between reported seizures and annotated ictal patterns in several patients (Table 2). For all three patients reporting daily absence seizures, we found daily spike-wave discharges that may reflect the reported absences. For four out of five patients who reported either myoclonic seizures, impaired awareness seizures or tonic-clonic seizures, we did not annotate ictal patterns. These seizures were possibly missed due to incomplete recordings, with some patients even notifying us that they did not wear the device during the seizure. As shown in Table 2, the compliance during the annotated period was suboptimal for the majority of patients who reported either myoclonic, impaired awareness or tonic-clonic seizures. All patients recorded more EEG during the day than during the night. For patient 4, reporting daily myoclonic seizures and three tonic-clonic seizures, we annotated daily polyspikes, daily spike-wave discharges and four (clusters of) ictal patterns. One of these clusters was not reported by the patient and occurred around 2 AM. We did not annotate ictal patterns for the three patients who did not report a seizure.

**Table 2:** **Comparison of reported seizures and annotated ictal patterns.** Compliances in the annotated period were calculated over the entire annotated period (first value) and only for days containing recorded data (value in brackets).

No.	**Reported seizures**	**Annotated ictal patterns**	**Compliance**
1	Daily absence seizures	Daily spike-wave discharges	88 % (88 %)
2	-	−	75 % (75 %)
3	Daily focal impaired awareness seizures	−	47 % (64 %)
4	Daily absence seizures	Daily spike-wave discharges	93 % (93 %)
	Daily myoclonic seizures	Daily polyspikes and spike-wave discharges	
	Three tonic-clonic seizures	Four (clusters of) ictal patterns	
5	-	−	79 % (79 %)
6	Two focal impaired awareness seizures	−	76 % (76 %)
7	Three myoclonic seizures	−	32 % (56 %)
	Six tonic-clonic seizures	−	
8	Eight tonic-clonic seizures	−	25 % (60 %)
9	-	−	91 % (91 %)
10	Daily absence seizures	Daily spike-wave discharges	26 % (44 %)

Some patients mentioned that the device occasionally lost connection during seizures, raising the possibility that EEG recordings during a seizure or post-ictally may not always be fully captured.

## 4 Discussion

This study presents our first experience with ultra-long-term two-channel sqEEG. Both electrode implantation and explantation were well tolerated, with a median VAS score of 4 and 2, respectively. Wearing the electrode for two and fifteen months were scored with a median VAS of 0 and 0.5, respectively. Despite the ongoing challenge of recording compliance, with a median of 24 %, a total of 28,331 h of data were collected by ten patients. We identified a circadian rhythm in ED rate in three patients and a circadian rhythm in ED duration in one patient.

### 4.1 Patient compliance

In 2019, the 24/7 EEG™ SubQ was used to record ultra-long EEG in nine epilepsy patients, achieving a compliance of 73 % (range 45–91 %), which is notably higher than the compliance in our study (27 %, range 1–74 %) [5]. However, their recordings lasted only up to three months. More recently, compliance rates between 88 and 95 % were achieved after three to six months of recording in five epilepsy patients [26]. Until now, the longest reported recording in an epilepsy patient had a duration of 230 days and a compliance of 86 % [8].

Other studies have recorded ultra-long sqEEG in healthy controls. A mean compliance of 64 % over 45 days was reported in twelve participants, with slight differences between daytime (66 %, range 29–89 %) and nighttime (61 %, range 3–94 %) compliance [27]. In 2024, sqEEG was recorded over the course of 365 nights in 25 participants, with 20 participants completing the study and achieving a mean compliance of 91 % (range 74–100 %) [28].

The relatively low compliance observed in our study may be attributed to several factors, including the fifteen-month recording period, inclusion of patients involved in the VNS process, epilepsy severity, and the absence of direct patient benefit. Besides, some other studies explanted the electrode when patients or participants indicated that they no longer wished to record [5,28]. In our study, however, we kept the electrode in place for the scheduled fifteen months and tried to motivate our patients to start recording again in the final month of the study, decreasing our compliances significantly. Finally, data were initially transferred via USB every two months, meaning that patients received feedback on their compliance once every two months. However, UNEEG medical A/S has recently implemented a cloud solution (MyConnect) for direct data transfer. This system, which is currently being used in the PREDYct study, enables more frequent compliance monitoring and more timely contact with patients when compliance declines. In addition, the system provides patients with feedback on their compliance after each upload, which may further encourage adherence. However, its long-term impact remains unknown, as none of our patients have used it for fifteen months, yet.

### 4.2 Epileptiform discharges

We identified a significant circadian rhythm in the ED rate in three out of ten patients. Additionally, three more patients exhibited a circadian rhythm in the ED rate, although not statistically significant. Previous studies have demonstrated that circadian rhythms in ED rate are a common phenomenon, although not universal among all epilepsy patients [[29], [30], [31]]. These rhythms are likely influenced by internal factors, including circadian fluctuations of hormone levels, along with external factors such as the wake-sleep cycle, eating habits and ASM administration [29]. We anticipate that with additional data and more annotations, we will be able to detect more than three significant circadian rhythms in the ED rate. The 1.5-day rhythm in ED rate observed in patient 10 may be attributed to significant variations in the patient’s sleep-wake cycle. A longer analysis period might uncover a more distinct circadian rhythm.

Some EDs may not have been annotated due to artefacts, missing data or EDs occurring in different brain regions, especially in focal epilepsy. Missing data may not follow a random pattern, complicating interpretation. For instance, patients might remove the device when feeling unwell, leading to a biased ED rate and duration, as moments with a potentially higher ED rate and/or ED duration go unrecorded. Additionally, if patients remove the device during the day but continue recording at night being unaware of their condition, this could introduce a bias in the circadian rhythms, highlighting the need for a high recording compliance. The compliance rates of patients 1 and 4 in the annotated period were 88 and 93 %, respectively, making bias unlikely to be a concern. Although more EEG was recorded during the day compared to the night, the low compliance of patient 7 (32 %) raises the possibility of bias in this case, and the identified circadian rhythm in ED rate for this patient should be interpreted with caution.

The comparison between seizure diaries and annotated ictal patterns demonstrated the necessity of high compliance for obtaining a reliable seizure count from sqEEG. Given that the compliance during the annotated period was suboptimal for most patients who reported either myoclonic, impaired awareness or tonic-clonic seizures, it is plausible that these patients were not recording sqEEG during the reported seizures. For patients with absence seizures, maintaining an accurate seizure diary was particularly challenging, as many were unable to recognize their seizures. This complicated the comparison of seizure diaries with sqEEG recordings for several patients. Furthermore, the lack of precise timestamps, with only the day of occurrence reported, represents a significant limitation of this study. Patients that are included in the PREDYct study are currently encouraged to maintain a more detailed seizure diary, including timestamps and the type of seizure experienced.

Our findings suggest that, in most cases, high compliance is required to reliably assess seizure frequency. For patients with multiple seizures per day, particularly those who are unaware of their seizures, sqEEG could be highly valuable, even with moderate compliance. However, for patients with less frequent seizures, higher compliance is needed to accurately track seizure frequency. Additionally, since sqEEG does not capture seizure type or patient-experienced burden, seizure diaries will likely continue to play an important complementary role in clinical practice.

In the period in which we manually annotated EDs (the first two weeks of recording), clear and distinct ictal patterns were identified in only one patient. Further, distinguishing IEDs from short absence and/or myoclonic seizures was challenging due to their overlapping features in several patients. Consequently, we decided not to perform an analysis solely focused on seizures.

In this study, we prioritized a subset of the data to ensure high-quality manual review and reliable assessment of interictal discharges and seizures. Given the immense, and practically impossible, time investment required for visual inspection of the total 15-month dataset, a fully automated approach is needed. However, current algorithms, including commercially available solutions, do not yet provide sufficient accuracy for IED detection or brief seizures in our data. This highlights the need for further development of a patient-specific automated ED detector, which constitutes the next phase of the PREDYct study.

Our primary focus in this study was on IEDs rather than seizures. While low compliance and incomplete seizure documentation limit the ability to draw definite conclusions about seizure frequency, this does not invalidate our analysis on interictal discharges. We show that sqEEG is feasible in patients undergoing VNS therapy, but we observed a large variability in compliance. We argue that this variability may, in part, result from the explorative character of this study as our patients do not directly benefit from the data recorded. However, the recordings may help identify biomarkers for optimizing VNS treatment, including assessing the likelihood of response to VNS, which is the primary goal of the PREDYct study.

## 5 Conclusion

We report our experiences with sqEEG in terms of patient experience and recorded EDs. Implantation and explantation of the electrode, as well as wearing the electrode for up to fifteen months were well tolerated. Recording compliance was a challenge (overall 27 %), with the lowest compliances at the end of the study period. Overall, 28,331 h of EEG were collected by ten patients. Three patients exhibited a circadian rhythm in ED rate and one patient showed a circadian rhythm in ED duration.

## Declaration of Generative AI and AI-assisted technologies in the writing process

During the preparation of this work the authors used ChatGPT in order to improve the wording and clarity of selected sentences. After using this tool, the authors reviewed and edited the content as needed and take full responsibility for the content of the published article.

## CRediT authorship contribution statement

**S.J. van Norden:** Writing – review & editing, Writing – original draft, Methodology, Formal analysis, Conceptualization. **K.H. Kho:** Investigation, Conceptualization. **A.M. Meppelink:** Supervision, Conceptualization. **J.J. Ardesch:** Supervision, Conceptualization. **M.C. Tjepkema-Cloostermans:** Writing – review & editing, Supervision, Methodology, Conceptualization. **M.J.A.M. van Putten:** Writing – review & editing, Supervision, Methodology, Conceptualization.

## Declaration of competing interest

The authors declare that they have no known competing financial interests or personal relationships that could have appeared to influence the work reported in this paper.
