# Supplementary material for: Ultra-long-term subcutaneous EEG recordings in ten epilepsy patients: Experiences and circadian rhythms in epileptiform discharges
Source: Epilepsy Behav Rep. 2025 May 20;31:100781. doi: 10.1016/j.ebr.2025.100781 (PMC12152861; doi:10.1016/j.ebr.2025.100781)

Supplementary material

In this supplementary material we present examples of annotated EDs for all patients. In addition, we show the ED rate and ED duration over the course of three days, including a Lomb-Scargle PSD (0.1% false-alarm probability in grey) and an overview of the circadian distribution based on the annotated data. Finally, the recording compliance is presented with sqEEG recordings in blue and both the start of the recordings and the explantation of the electrode in pink.

Patient 1

Gender:	Female	Recorded data:	2338 hours (compliance of 20%)
Age:	20-24 years	Annotated data:	283 hours
Type of seizures:	Generalized	Annotated EDs:	1943 EDs
Implantation side:	Right		

EDs

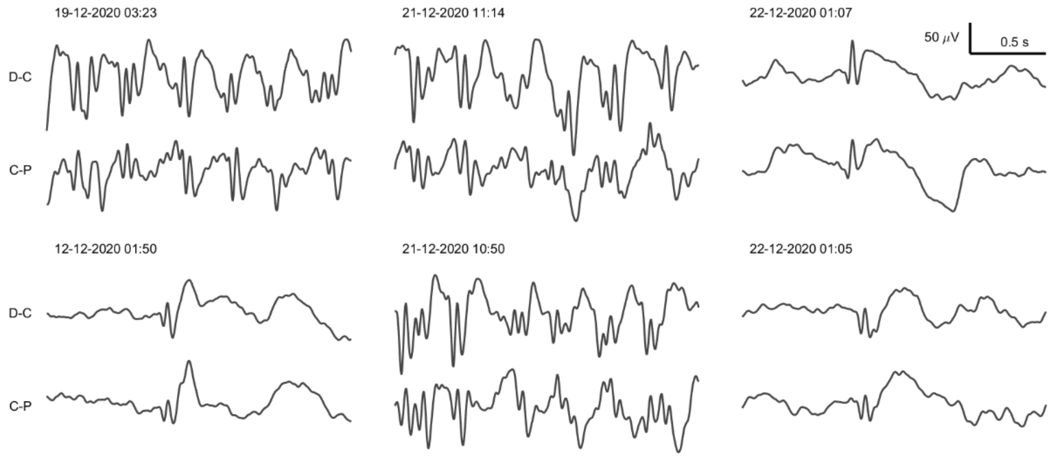

ED rate and ED duration

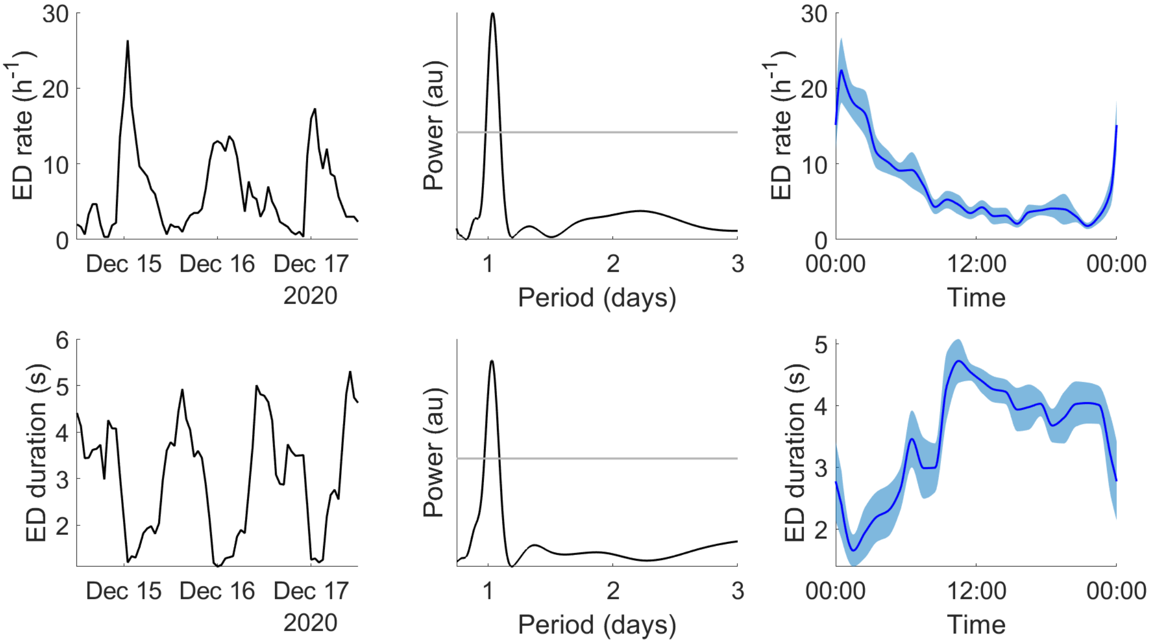

Compliance

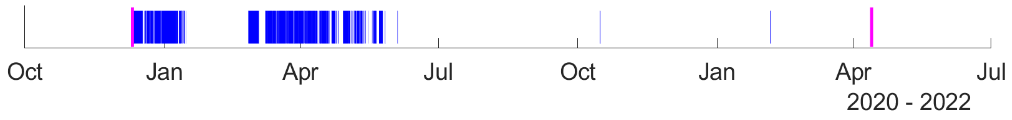

Patient 2

Gender:	Female	Recorded data:	3146 hours (compliance of 27%)
Age:	20-24 years	Annotated data:	245 hours
Type of seizures:	Generalized	Annotated EDs:	105 EDs
Implantation side:	Left		

EDs

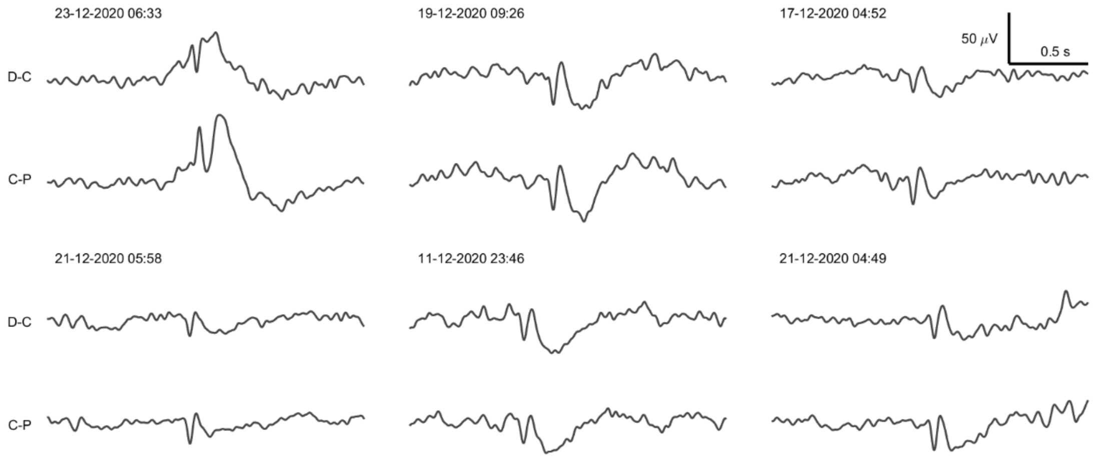

ED rate and ED duration

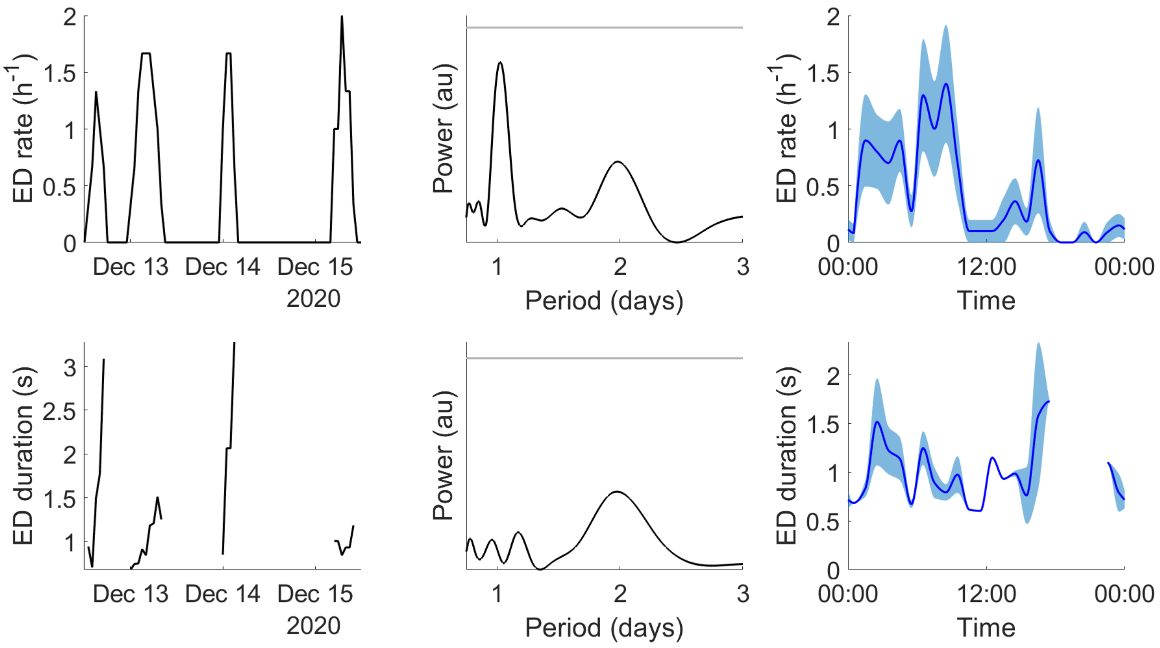

Compliance

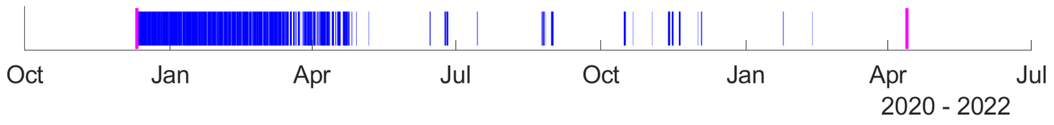

Patient 3

Gender:	Male	Recorded data:	2176 hours (compliance of 21%)
Age:	40-44 years	Annotated data:	263 hours
Type of seizures:	Generalized & focal	Annotated EDs:	41 EDs
Implantation side:	Left		

EDs

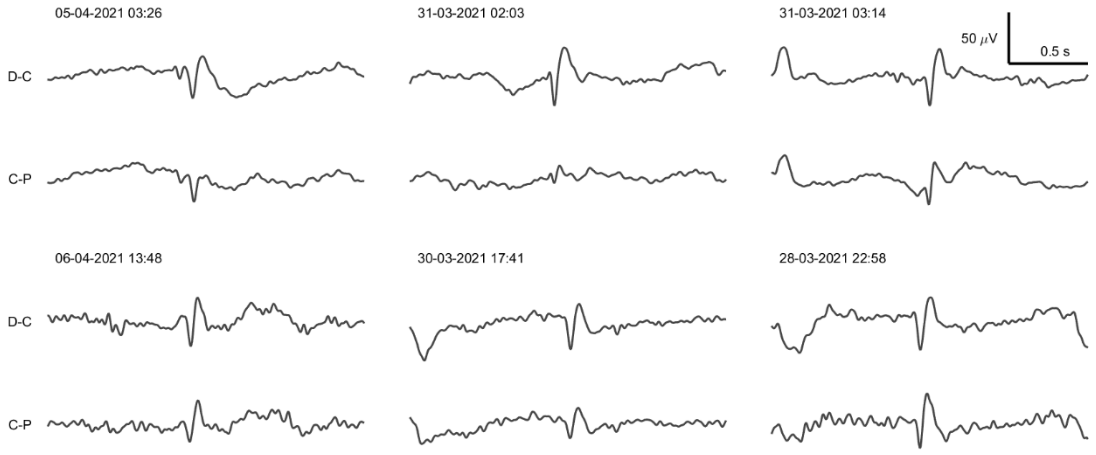

ED rate and ED duration

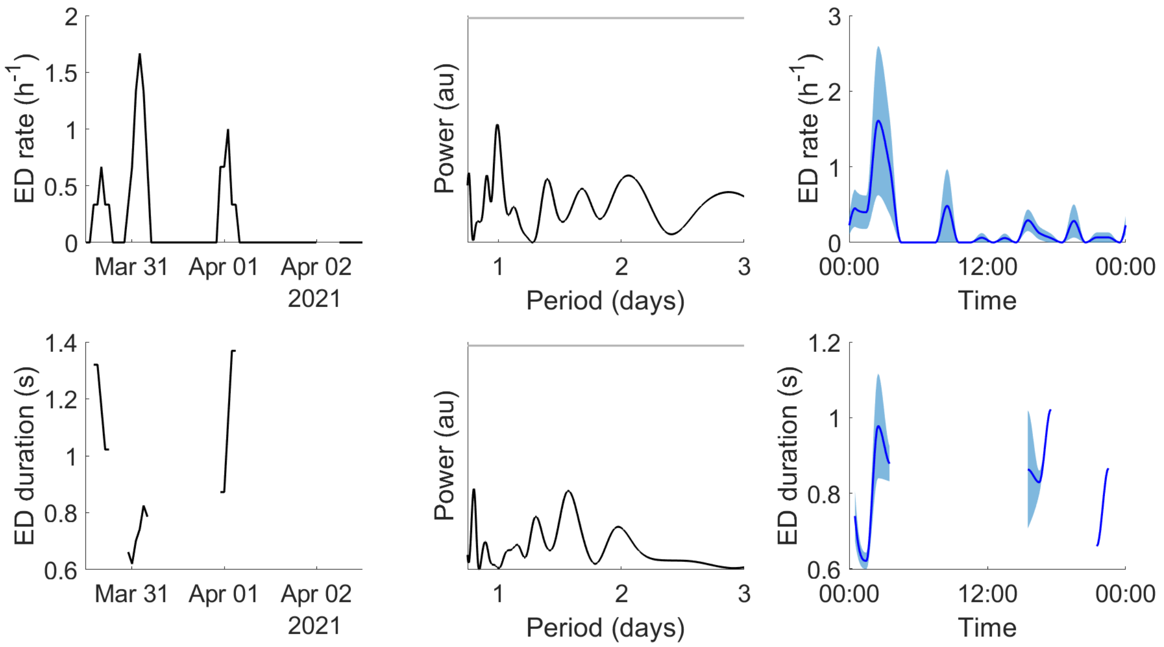

Compliance

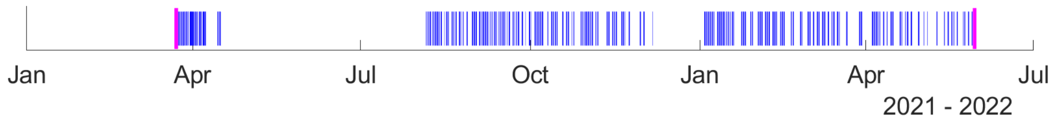

Patient 4

Gender:	Male	Recorded data:	2846 hours (compliance of 34%)
Age:	50-54 years	Annotated data:	348 hours
Type of seizures:	Generalized	Annotated EDs:	3144 EDs
Implantation side:	Left		

EDs

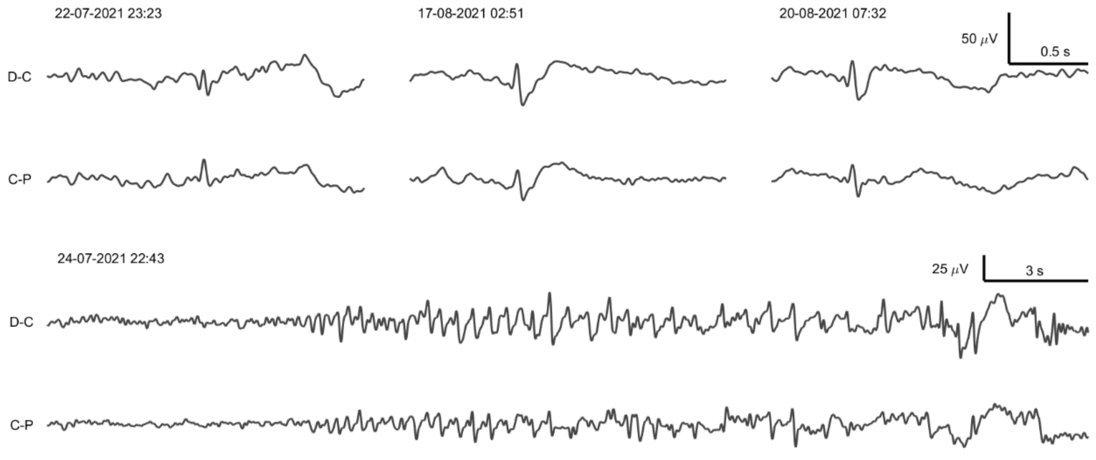

ED rate and ED duration

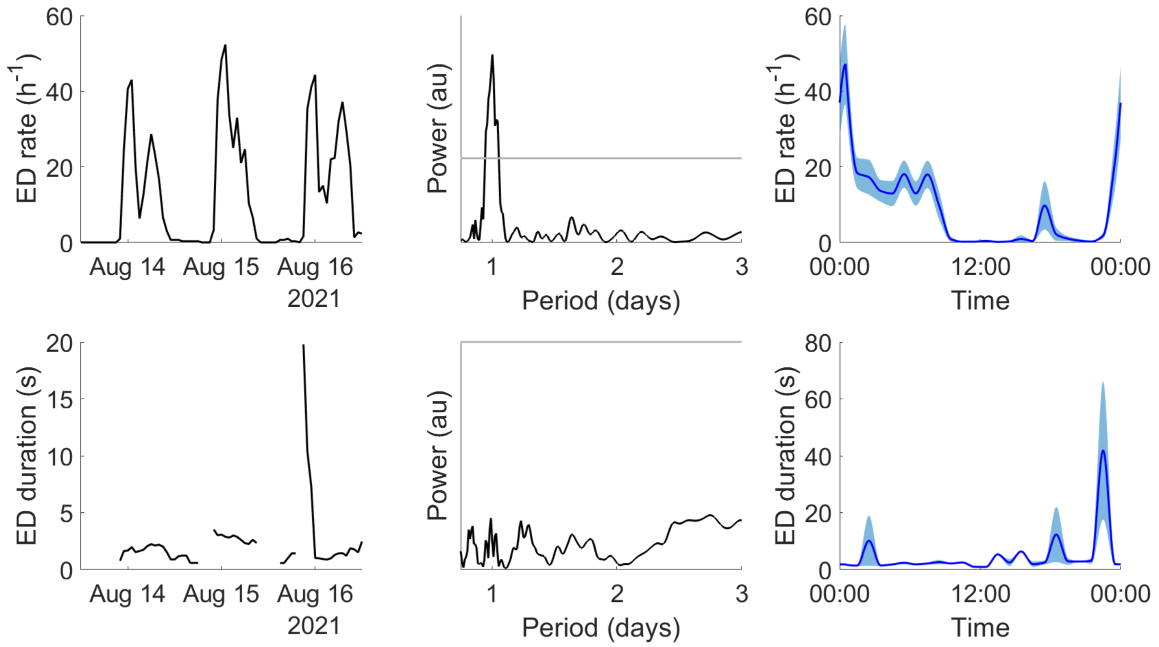

Compliance

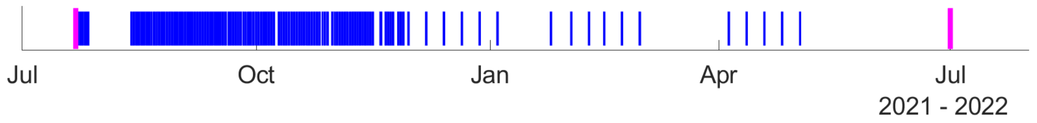

Patient 5

Gender:	Female	Recorded data:	150 hours (compliance of 2%)
Age:	25-29 years	Annotated data:	150 hours
Type of seizures:	Focal	Annotated EDs:	32 EDs
Implantation side:	Right		

EDs

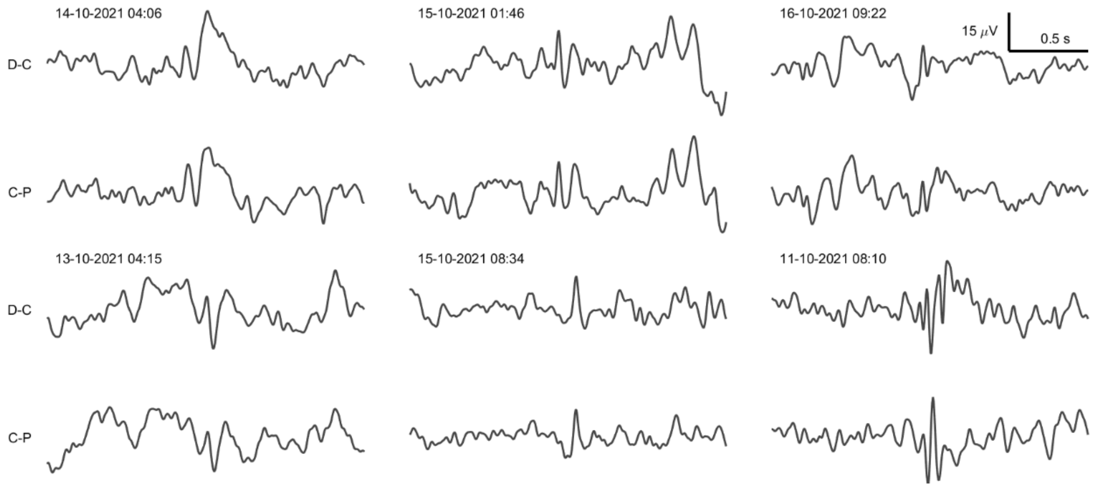

ED rate and ED duration

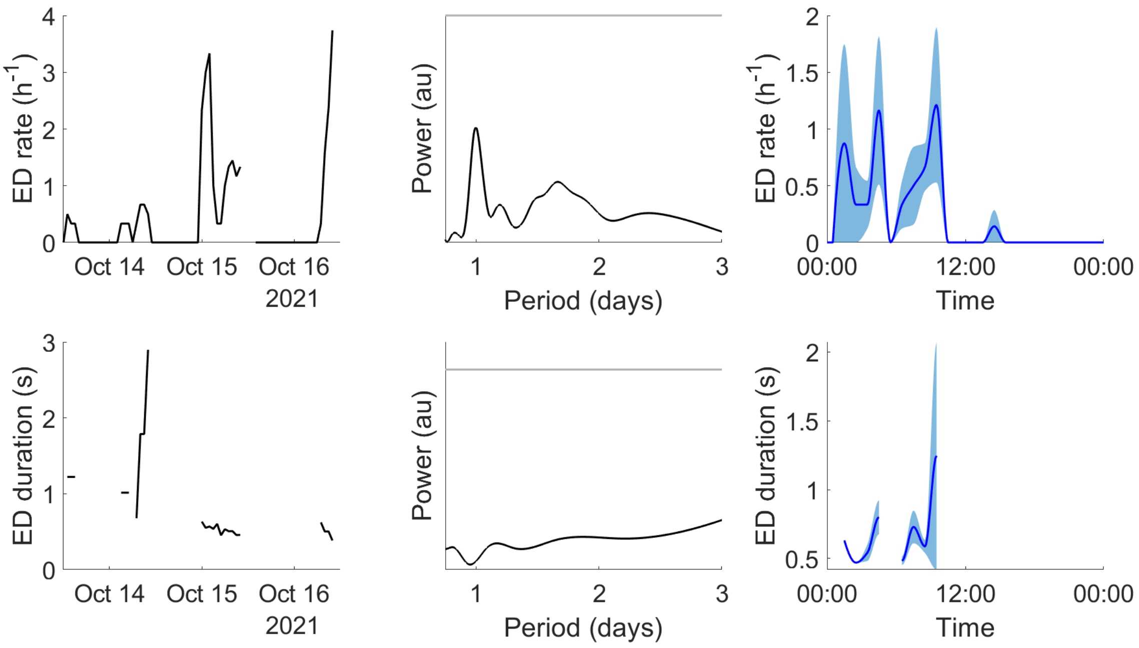

Compliance

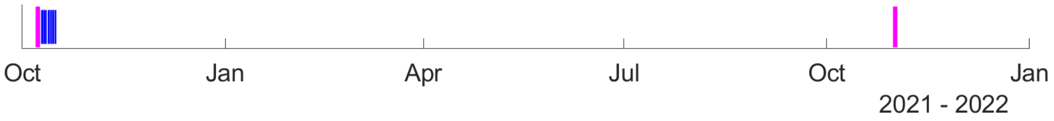

Patient 6

Gender:	Male	Recorded data:	6365 hours (compliance of 61%)
Age:	50-54 years	Annotated data:	235 hours
Type of seizures:	Focal	Annotated EDs:	5 EDs
Implantation side:	Left		

EDs

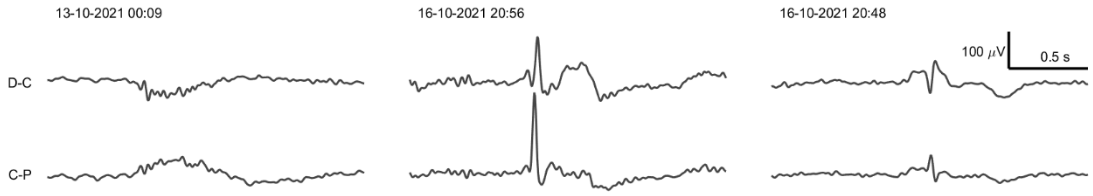

ED rate and ED duration

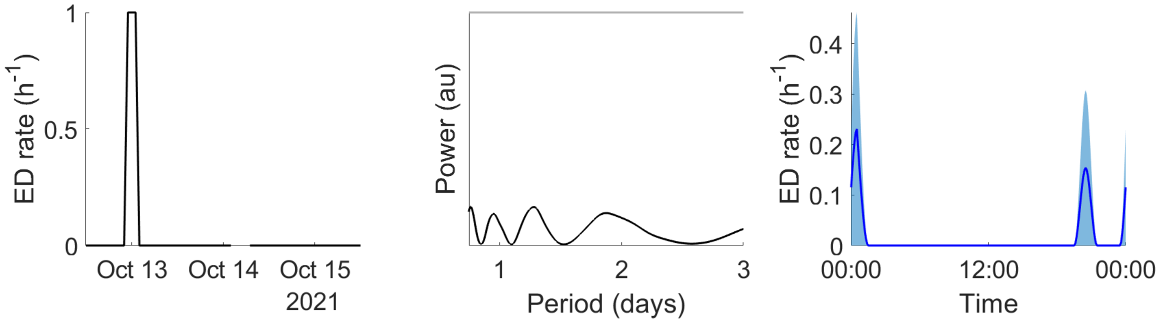

Compliance

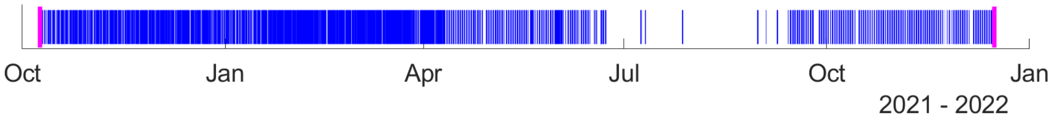

Patient 7

Gender:	Male	Recorded data:	172 hours (compliance of 1%)
Age:	35-39 years	Annotated data:	167 hours
Type of seizures:	Generalized	Annotated EDs:	551 EDs
Implantation side:	Left		

EDs

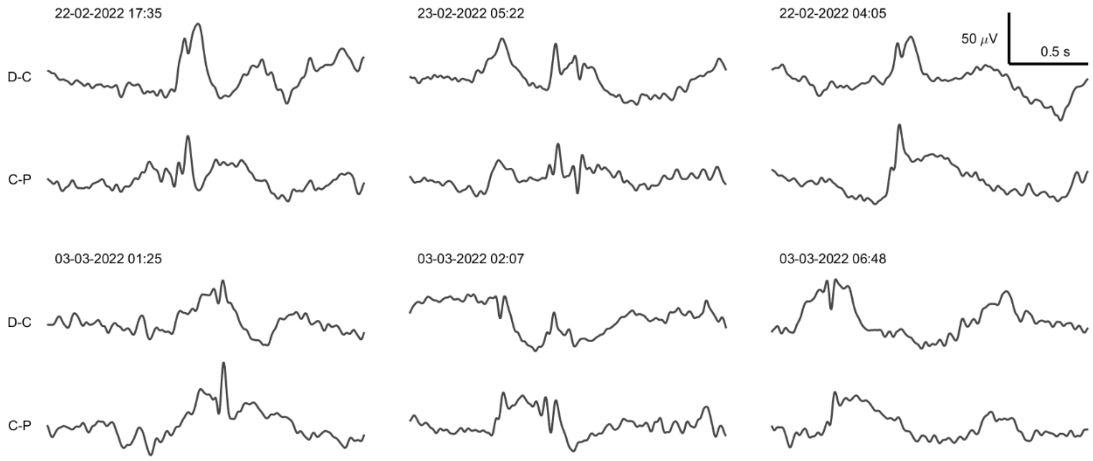

ED rate and ED duration

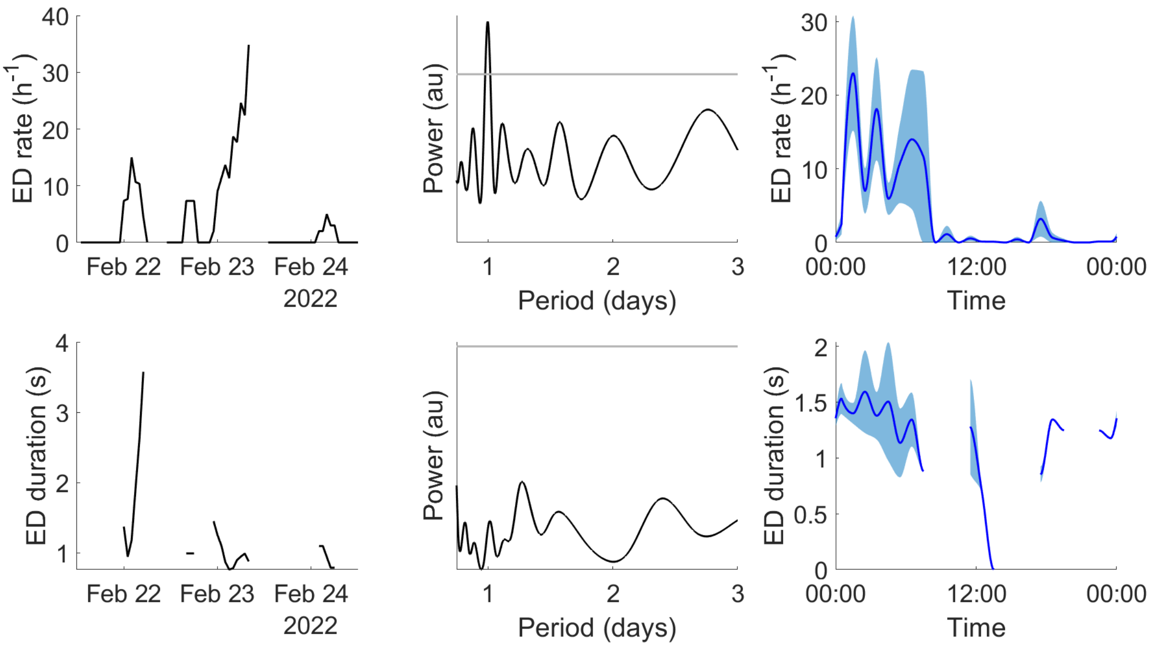

Compliance

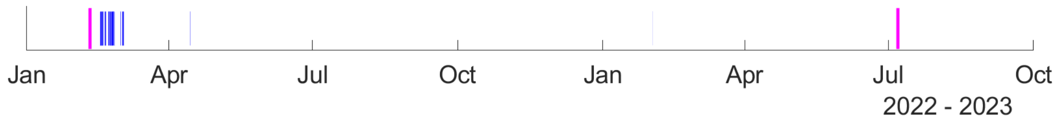

Patient 8

Gender:	Female	Recorded data:	2863 hours (compliance of 27%)
Age:	30-34 years	Annotated data:	228 hours
Type of seizures:	Generalized	Annotated EDs:	8 EDs
Implantation side:	Right		

EDs

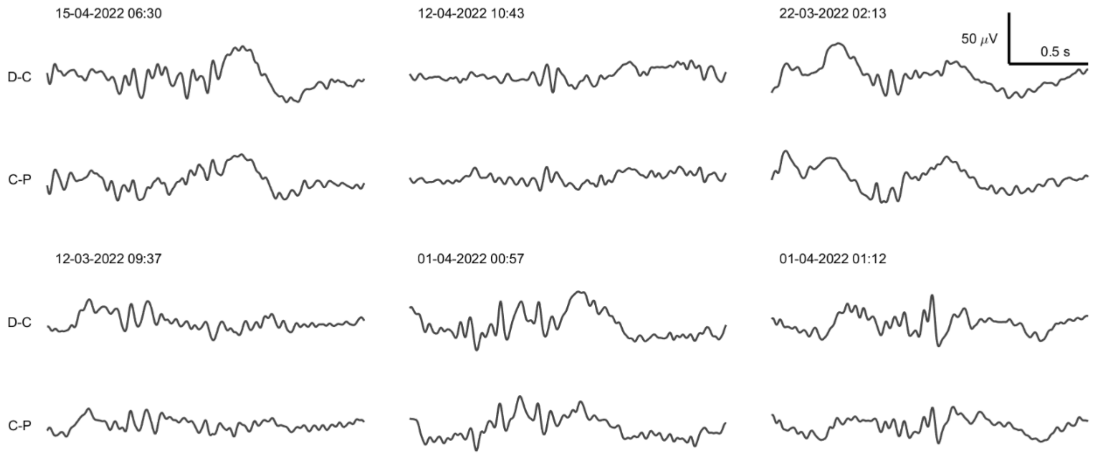

ED rate and ED duration

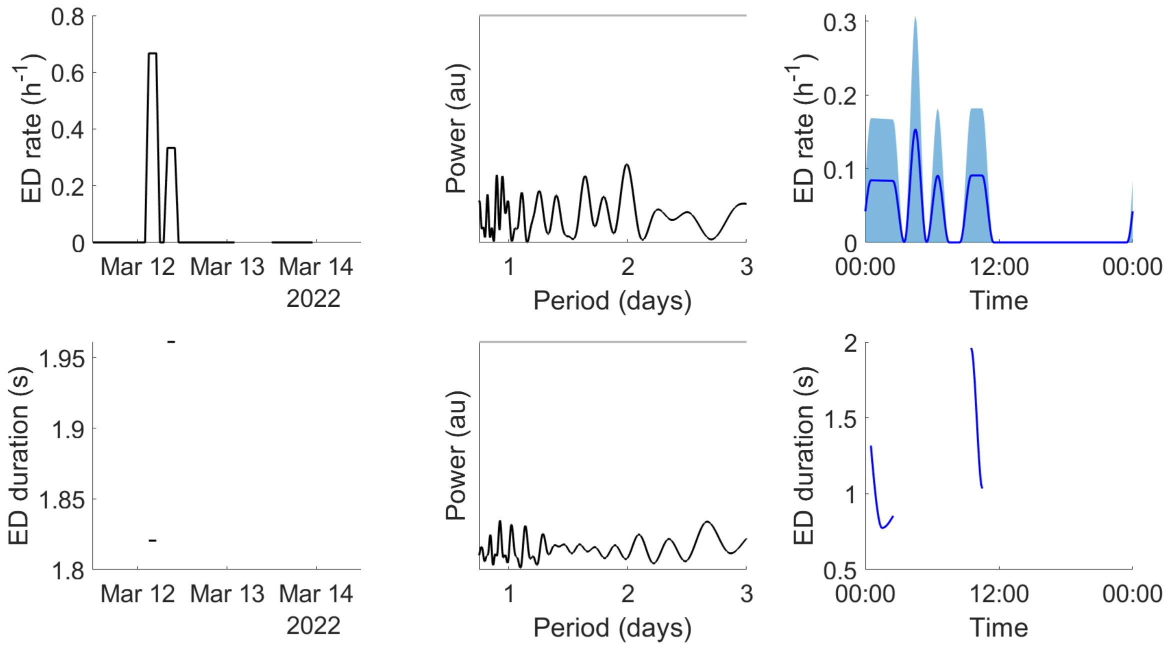

Compliance

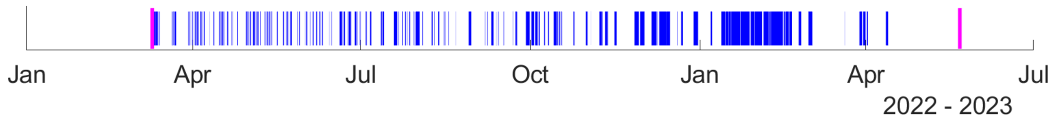

Patient 9

Gender:	Female	Recorded data:	7878 hours (compliance of 73%)
Age:	35-39 years	Annotated data:	375 hours
Type of seizures:	Focal	Annotated EDs:	54 EDs
Implantation side:	Right		

EDs

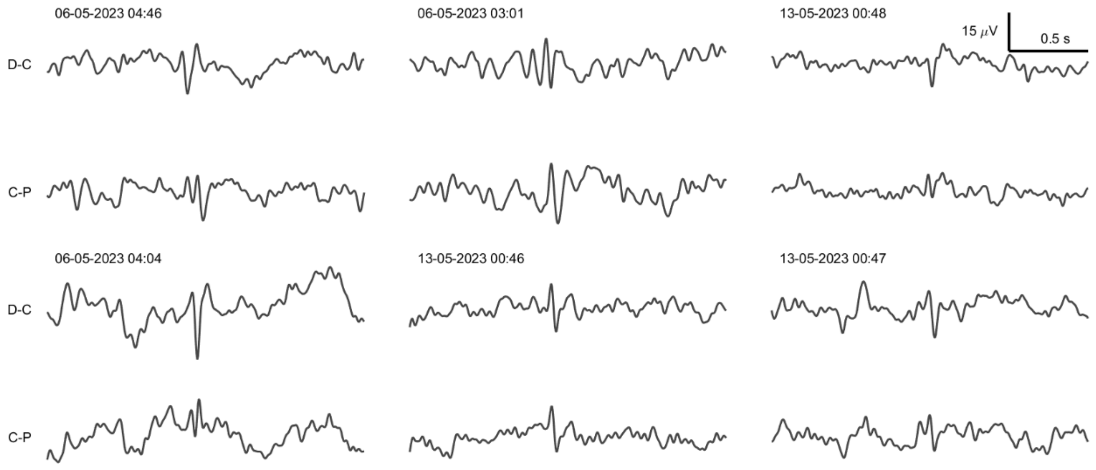

ED rate and ED duration

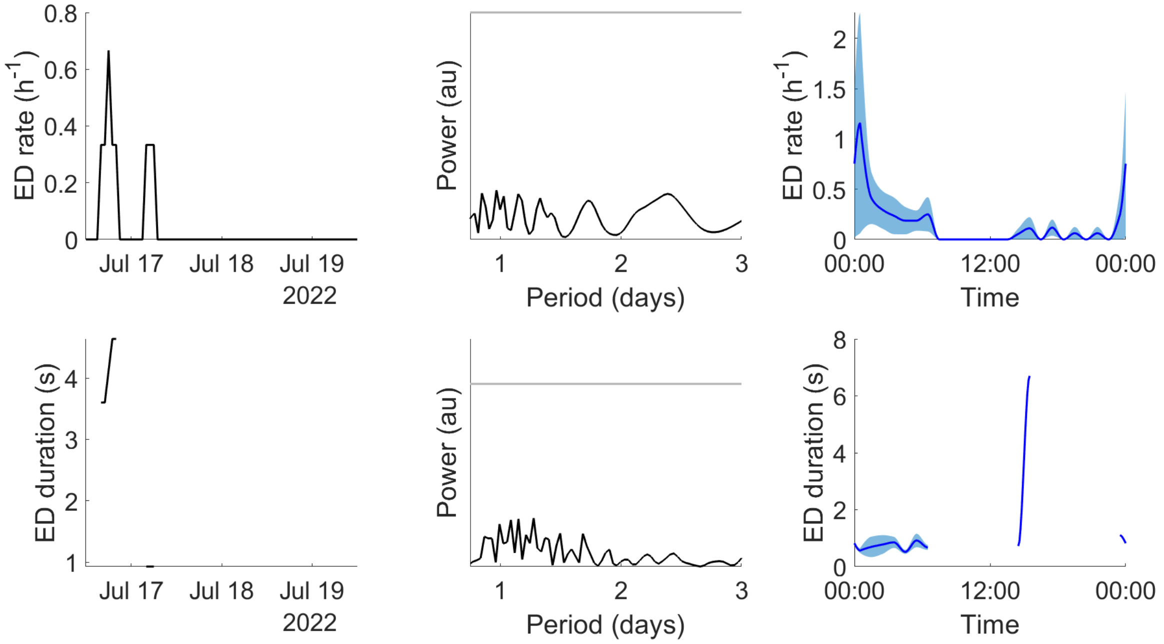

Compliance

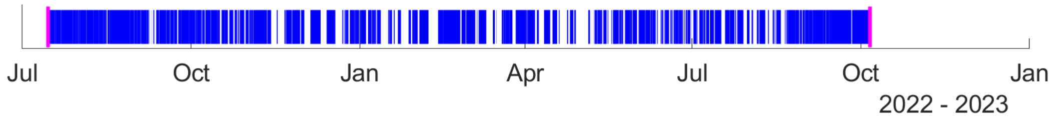

Patient 10

Gender:	Male	Recorded data:	398 hours (compliance of 4%)
Age:	20-24 years	Annotated data:	242 hours
Type of seizures:	Generalized	Annotated EDs:	2508 EDs
Implantation side:	Right		

EDs

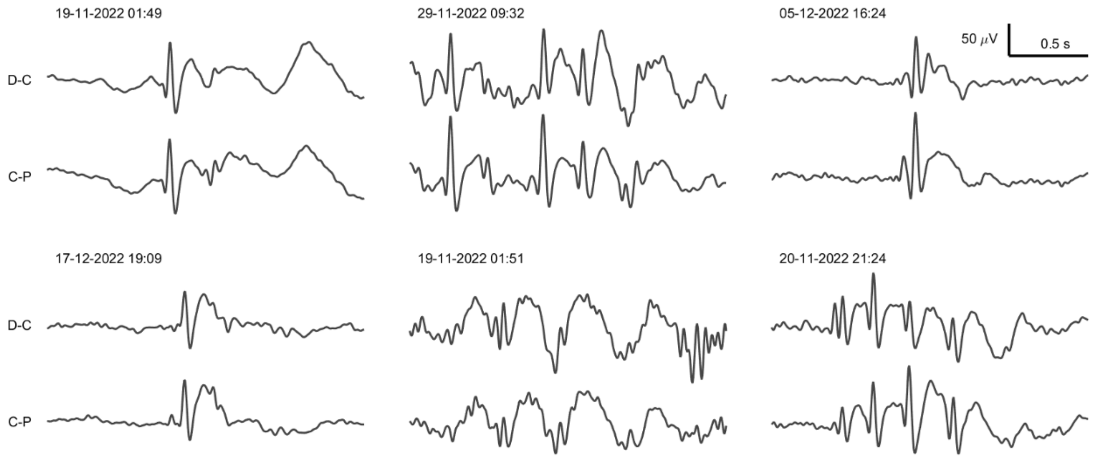

ED rate and ED duration

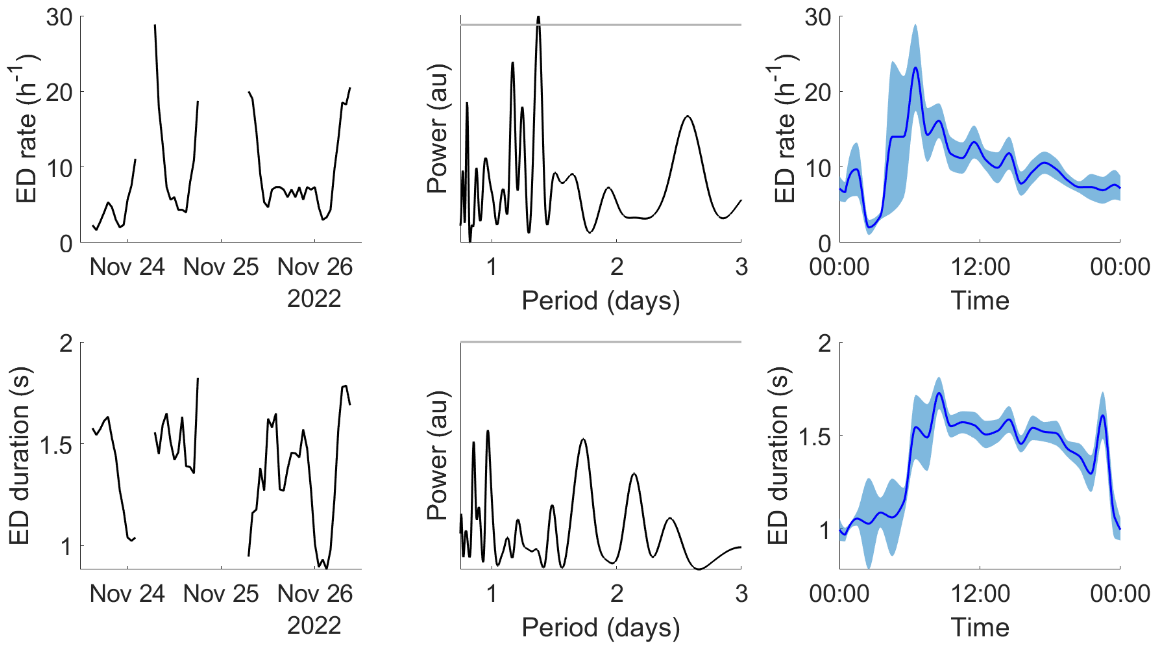

Compliance

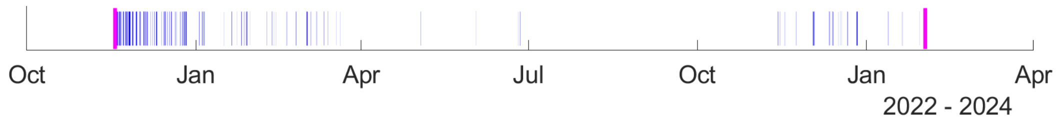

Supplement: Supplementary data 1 [file mmc1.pdf]
